# Barriers and facilitators to implementing digital psychosocial interventions for older adults presenting to emergency departments: a scoping review

**DOI:** 10.1186/s12913-026-14129-6

**Published:** 2026-02-19

**Authors:** Neve Davison, Alex Waddell, Anu Ivaturi, Dharshani Chandrasekara, Thach Tran, Maggie Kirkman, Karin Hammarberg, Seema Nimesh, Judy Lowthian, Patrick Olivier, Lorena Romero, Jane Fisher, Rosamond Dwyer

**Affiliations:** 1 Global and Women’s Health, School of Public Health and Preventive Medicine, Monash University, Level 4, 553 St Kilda Rd, Melbourne, VIC 3004 Australia; 2 Action Lab, Department of Human Centred Computing, Monash University, Melbourne, VIC Australia; 3 Emergency Department, Peninsula Health, Frankston, VIC Australia; 4 School of Public Health and Preventive Medicine, Monash University, Melbourne, VIC Australia; 5 The Ian Potter Library, The Alfred, Melbourne, VIC Australia

**Keywords:** Older adults, Hospital emergency services, Psychosocial care, Digital health

## Abstract

**Background:**

Globally, we are experiencing an ageing population. In high-income countries, many individuals aged over 60 years live with at least one chronic condition. Among older people, certain psychosocial factors are associated with increased risk of experiencing an emergency department (ED) presentation. These presentations may have negative physical and psychological outcomes for individuals and place additional burdens on health resources. The implementation of digital psychosocial interventions could help create age-friendly EDs and improve holistic healthcare provision, reduce re-presentations and better address the challenges faced by older adults. This scoping review aimed to identify and synthesise the barriers and facilitators to implementing digital psychosocial interventions designed for older adults to assess or manage non-primary psychosocial complaints in ED.

**Methods:**

Four databases and Google Scholar were searched for experimental, observational and qualitative peer-reviewed studies or grey literature published in English. Papers were considered for inclusion if they described the perspectives of key stakeholders (older adults, family, or healthcare providers) on the implementation of digital psychosocial interventions designed for older adults in emergency settings. Study selection and data extraction were performed in duplicate, with the Theoretical Domains Framework used to synthesise barriers and facilitators.

**Results:**

Eight papers from four countries met the eligibility criteria. They employed qualitative and/or quantitative methods and reported on digital interventions addressing various psychosocial needs. Older adults identified the highest number of barriers and facilitators, followed by healthcare providers, with families and caregivers reporting the fewest. The barriers recognised by older adults included a lack of technological skills and a fear or aversion to technology, while facilitators were improved privacy and designing digital psychosocial interventions to enhance user experience. Healthcare professionals faced barriers due to ED’s demanding environment and heavy workloads. However, supportive leadership was noted as a facilitator for implementation. Families and caregivers were the least involved stakeholders, resulting in very few barriers or facilitators being identified in this group.

**Conclusions:**

The barriers and facilitators identified in this review highlight that there is promising evidence to support the implementation of digital psychosocial interventions that are co-designed with older adults, families and clinicians to improve care and reduce workloads.

**Supplementary Information:**

The online version contains supplementary material available at 10.1186/s12913-026-14129-6.

## Background

Older adults (aged 60 + years) constitute one-eighth of the population but account for more than one-quarter of emergency department (ED) admissions globally [1–4]. The world’s population is ageing rapidly; by 2050, the number of older adults in high-income countries is expected to account for over one-third of the population [5]. Compared to younger adults, older adults attend EDs more frequently and have an increased likelihood of repeat visits [3, 6]. Older adults often have complex health needs that are distinct from those of younger people [7–9]. Clinical presentations may be complicated by multiple comorbid conditions and non-specific symptoms [3, 6, 10]. Frequent and potentially avoidable ED presentations may contribute to strain on scarce healthcare resources.

Good psychosocial care is a possible strategy to reduce risk of ED visits by older adults [11]. Recent data from a prospective study of approximately 11,000 older Australians identified that depressive symptoms in otherwise healthy older adults increased the likelihood of admission to the ED within the next three years [9]. This has been shown in other countries in which older adults with symptoms of anxiety and depression who have acute hospital admission have higher rates of hospital readmission [12, 13]. Older adults are more likely to experience events such as bereavement, change to socioeconomic status due to retirement, social isolation and loss of independence [14, 15]. These factors can contribute to poor mental health and additional psychosocial support and care needs [14, 15]. Older people presenting to hospital with depression and loneliness have been found to report a high frequency of non-specific symptoms such as chest pain, fatigue, back pain and dizziness [16–18]. These symptoms may complicate the initial assessment and make it difficult for clinicians to identify the underlying condition [19]. However, significant gaps remain in the assessment of psychosocial factors and common mental health conditions in the ED [20]. 

Digital health tools are used for prevention, health promotion, diagnosis, referral processes, monitoring and improving access to health information or healthcare services [21]. There has been a proliferation of digital health tools in recent years; for example, most tertiary health services in high-income countries have implemented electronic medical record systems [22]. In ED settings, digital tools have included patient assessments on portable electronic devices, ED information systems, clinical decision support systems, telemedicine, electronic health records, and personal health records [23]. These tools have shown the potential to improve workflow and quality of care, as well as reduce data collection-related burdens for clinicians [22]. However, the use of digital tools for older adults necessitates a nuanced understanding of their specific needs.

Older adults are generally accepting of and interested in the use of digital health technologies [24], potentially facilitating their implementation and uptake in EDs. However, older adults are not a homogeneous group, and different factors influence the uptake of digital health technology across this population [25]. Emphasising the need for and benefits of specific digital health technologies for older adults has been shown to positively influence uptake [26]. However some older adults encounter technical challenges and lack self-efficacy [27, 28], which limits the applicability of these tools. Additionally, research has shown that ageism and biases among healthcare workers may inhibit uptake of digital health technology [26]. To address factors affecting implementation, digital health technologies should be co-designed with end-users (clinicians, older adults, their families and carers) [27, 28]. 

Implementing digital tools in EDs requires infrastructure, workflows, staff and patient behaviour changes [29]. Given the varied stakeholder groups involved in older adults’ care in EDs (including patients, clinicians, family members, and administrative staff), it is crucial to consider the barriers and facilitators to implementation from diverse perspectives [30–32] and how environmental, contextual and system-level factors influence implementation. The aim was to identify the factors that act as barriers and facilitators to implementing digital psychosocial interventions designed for older adults in EDs from various stakeholder perspectives.

### Review question

What are the barriers and facilitators to implementing digital psychosocial interventions for older adults in EDs?

## Methods

This scoping review was conducted and reported following the Joanna Briggs Institute (JBI) [33] guidelines and the Preferred Reporting Items for Systematic Reviews and Meta-Analyses extension for Scoping Reviews (PRISMA-ScR) checklist [34]. Scoping review methods were followed as they allowed for a broad scope of all relevant literature. The protocol of this scoping review has been published [35]. 

### Protocol deviation

Due to a dearth of studies investigating the barriers and facilitators to implementation of digital psychosocial technologies designed for adults older than 70 years, we modified this eligibility criterion from that outlined in the protocol [35] to include adults 60 years and older.

### Search strategy

A comprehensive search strategy was developed in consultation with a specialist information analyst (LR). Search terms included relevant keywords and subject headings and covered three key concepts: emergency department, digital health technologies, and older adults (see appendix 1 for an example search on Medline). Four databases, Medline (OVID), Embase (OVID), PsycINFO (OVID) and SCOPUS were searched for relevant articles from database inception until June 2024. An additional search strategy was developed for Google Scholar, where the first 100 results were screened for relevant grey literature or other documents to be included in this scoping review. Forward and backward citation searching was also conducted to identify any additional relevant articles.

### Eligibility criteria

The eligibility criteria were developed using the Sample, Phenomenon of Interest, Design, Evaluation and Research type (SPIDER) framework [36]. Inclusion and exclusion criteria are shown in Table 1.

**Table 1:** SPIDER eligibility criteria

SPIDER Element	Inclusion	Exclusion
Sample	• Adults aged ≥ 60 years receiving care in emergency department settings • Stakeholders involved in the implementation, delivery, or use of digital psychosocial interventions (e.g., families, healthcare providers, administrators, non-clinical leaders)	• Adults aged < 60 years
Phenomenon of Interest	• Digital psychosocial interventions designed for or used with older adults and implemented within emergency department settings (e.g., applications on portable electronic devices, digital screening tools, and computer-delivered questionnaires)	• Non-digital interventions • Multimodal interventions • Telehealth or telemedicine • Remote patient monitoring
Design	• Interviews • Focus groups • Workshops • Surveys • Case studies • Observational studies • Randomised control trials • Grey literature • Conference proceeding papers	• Letters • Editorials • Protocols • Proof of concept studies • Feasibility studies • Systematic reviews
Evaluation	• Experiences • Views • Attitudes • Perceptions • Knowledge related to implementation	• Studies reporting effectiveness outcomes only • Intervention development without implementation data
Research Type	• Qualitative • Quantitative • Mixed methods	

### Study selection

All records identified through database and Google Scholar searches were uploaded to a purpose-built screening platform, Covidence (Veritas Health Innovation, Melbourne, Australia) [37]. Papers were screened and evaluated for inclusion through a two-stage process. First, titles and abstracts were screened independently by three authors (ND, AI, DC). Any disagreements regarding the inclusion of a paper were resolved through discussion until consensus was reached, or the second author (AW) was consulted to determine eligibility.

This same process was used for the second stage, where the first and third authors (ND and AI) independently reviewed the full text of each paper identified in the first stage and decided if papers met the inclusion criteria; if they did not, a reason for exclusion was provided.

### Data extraction

Following study selection, the first and third authors (ND and AI) used piloted data extraction forms to extract data in Covidence and Microsoft Excel. Data extracted for each included paper were article reference, country of origin and language of the publication, study objectives and design, conceptual or theoretical framework, participant characteristics (e.g., number, age, methods of recruitment), living situation (whether participants were living in the community or residential aged care), methodological approach, start and end dates to study, funding sources, whether ethics approval was secured, digital intervention description, description of psychosocial factor/s and description of the environment in which the intervention was implemented. Outcome data extracted were barriers and/or facilitators to implementation, timing of outcome measurement (e.g., pre- or post-implementation), and study limitations (author reported). Methodological limitations were assessed using an appropriate checklist from the CASP [38]. 

Data extracted from qualitative studies were details of data collection methods (e.g., semi-structured interviews, focus groups, observations). Outcome data from qualitative studies were captured as quotations and author-reported themes, categorised as either barriers or facilitators to implementation. A similar process was used when extracting quantitative data on barriers and facilitators.

### Data synthesis

Following data extraction, data synthesis was performed using the ‘best-fit framework synthesis’ approach [39] as recommended by The Cochrane Qualitative Review Methods Group [40]. Two authors (ND and AI) independently deductively coded the extracted data into the Theoretical Domains Framework (TDF) using NVivo V.14. The TDF [29] is a behaviour change framework with 14 domains for organising the factors (at the individual, organisational, and system levels) that influence behaviour and inform behaviour-change interventions. The TDF facilitates synthesis across various perspectives, highlighting where factors do and do not align [41]. Quotations (or when quotations were unavailable, author interpretations) and empirical data, as well as author conclusions were coded against the 14 domains of the TDF. If codes did not match, the two reviewers discussed until consensus. Anything not coded deductively to the TDF domains was coded inductively to identify any additional themes. The numbers of barriers and facilitators were assessed as frequency per study. Each barrier or facilitator was counted only once per study. Where studies reported several stakeholder perspectives, barriers and facilitators were counted once per stakeholder group.

## Results

**Fig. 1:**
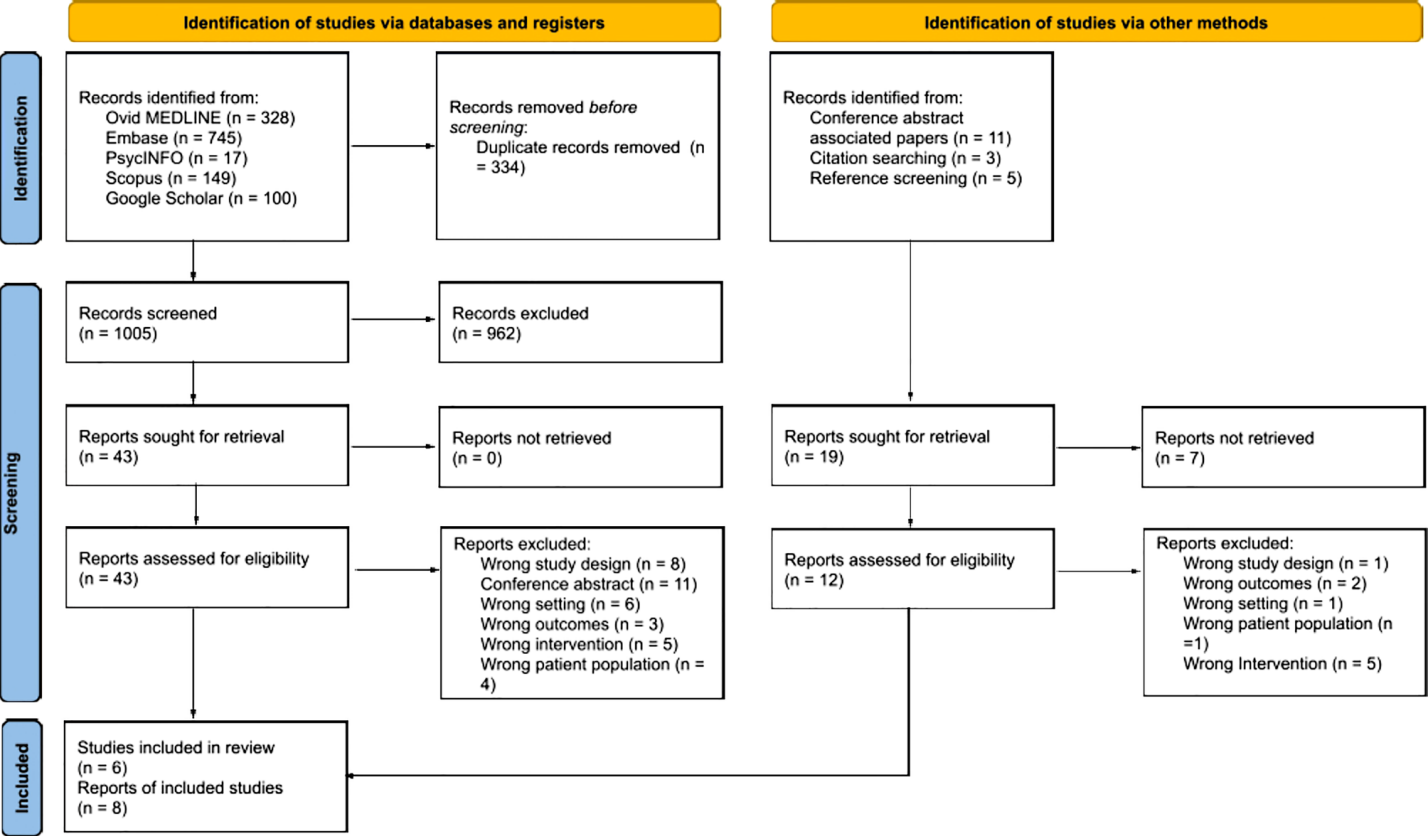
PRISMA flow diagram. *From*: Page MJ, McKenzie JE, Bossuyt PM, Boutron I, Hoffmann TC, Mulrow CD, et al. The PRISMA 2020 statement: an updated guideline for reporting systematic reviews. BMJ 2021;372:n71. 10.1136/bmj.n71. For more information, visit: http://www.prisma-statement.org/

The four databases and Google Scholar yielded a total of 1005 citations. Of these, 43 underwent full-text assessment. Thirty-seven articles were excluded for various reasons (see Appendix 2). Of the 11 conference abstracts excluded, the full-text of four associated studies were retrieved and assessed for inclusion. One was excluded for ineligible study design and one for reporting the wrong outcomes. As a result, eight publications reporting on six studies were found to be eligible and were included in this scoping review (Fig. 1). After consulting the authors, we found that Abujarad et al. (2021) [42] and Choo et al. (2021) [43] reported on focus groups from the same study. For this reason, only quantitative data from Abujarad et al. (2021) [42] were extracted and analysed. Qualitative data were extracted and analysed only from Choo et al. (2021) [43] because this was a more complete data set. Boucher et al. (2021) [44] reported a sub-analysis of the data collected by Boucher et al. (2019) [45].

Forward and backward screening were then performed to ascertain if any additional studies could be included in this scoping review. An additional eight studies were identified in this process, but all were excluded for the following reasons: wrong intervention (*n* = 5), wrong setting (*n* = 1), wrong patient population (*n* = 1), and not reporting barriers and facilitators to implementation (*n* = 1). Figure 1 depicts the PRISMA flow diagram for study selection.

### Study characteristics

This review comprised eight reports of six studies. Of these papers, four used quantitative methods, two used qualitative methods and two used mixed-methods. The sample sizes ranged from 14 to 488. Of the six studies, two were conducted in the United States of America, two in Canada, one in Finland and one in Iran. The psychosocial concerns targeted by the digital health technologies investigated in the studies were cognitive function (*n* = 4), functional status (*n* = 2) frailty (*n* = 2), elder abuse (*n* = 1), falls risk (*n* = 1), nutrition risk (*n* = 1) and general care of older adults (*n* = 1). The interventions included digital health tools for screening, one in the format of a serious game (a game that has a purpose beyond entertainment such as education, rehabilitation and medical care [46, 47]) on a touch-screen tablet. For example, Tong et al. (2016) [48] used a serious game to facilitate a cognitive assessment. Three [42, 48, 49] of the six studies reported the use of digital devices by their older adult populations. Five of the six studies only recruited older adult participants living in the community; Boucher et al. (2019) [45] also recruited those living in residential aged care. Table 2 details the characteristics of each study.

**Table 2:** Characteristics of included studies

Author (Year) Location	Study Design; method	Population Description (*n*, mean age)	Psychosocial factor/s	Intervention
**Mixed Methods Studies**				
^a^Abujarad et al. (2021) USA [42]	Focus groups & usability survey	Focus groups: 60 + years community-dwelling & cognitively intact, clinicians and caregivers (24, 70); Usability: 60 + years community-dwelling & cognitively intact (14, 71.4)	Elder abuse	Screening tool on tablet
**Quantitative Studies**				
Brahmandam et al. (2016) USA [49]	Cross sectional study	65 + years community-dwelling receiving care in two academic EDs (248, NR)	Cognitive status	Screening tool on tablet
^b^Boucher et al. (2019) Canada [45]	Randomised cross-over pilot trial	65 + years community- or residential aged care-dwelling ED patients and caregiver if present (67, 75.5)	Functional status, cognitive status, frailty	Screening tool on tablet
^b^Boucher et al. (2021) Canada [44]	Randomised cross-over pilot trial	65 + years community- or residential aged care-dwelling ED patients and caregiver if present (60, 74.4)	Functional status, cognitive status, frailty	Screening tool on tablet
Saario et al. (2021) Finland [50]	Observational study	70 + years community-dwelling (488, 82.8)	Cognitive status, falls risk, nutrition risk	Screening tool on tablet
**Qualitative Studies**				
^a^Choo et al. (2021) USA [43]	Focus groups	60 + years community-dwelling & cognitively intact, clinicians and caregivers (24, 71)	Elder abuse	Screening tool on tablet
Shagerdi et al. (2022) Iran [51]	Interviews	geriatricians, geriatric nurses, emergency medicine specialists & emergency department nurses (33, N/A)	general care for older adults	No specific intervention
Tong et al. (2016) Canada [48]	Survey	65 + years community-dwelling call 911 or presented to ED (325, 75.8)	Functional status, cognitive status, frailty	Screening tool on tablet; serious game

a, b represents reports on the same study, N/A = not applicable, NR = not reported

### Quality assessment

The heterogeneity of the included studies required various quality appraisal checklists to be used. All but one paper [49] reported receiving ethics approval; no response to a request for further information was obtained from the authors.

The quality of studies using qualitative and mixed methods was moderate to high. All included studies had clear aims, no apparent failures to address ethics, and a clear statement to report findings (Appendix 3). Most of the qualitative and mixed-methods papers adequately reported the relationship between researchers and participants and used an appropriate study design to answer their study aims. However, it was unclear whether Abujarad et al. (2021) [42] reported on the same focus groups as Choo et al. (2021) [43], necessitating direct contact with the authors. One qualitative study was of low quality as it did not use a transparent and replicable recruitment strategy or data collection methods, nor did it identify the relationship between researchers and participants [51]. 

Of the included quantitative studies, there was one randomised pilot trial, one cross-sectional study and one cohort study. The randomised pilot trial [45] was of low quality (as determined by the CASP checklist) as it did not include participant or assessor blinding, and the findings have limited external validity (Appendix 3). The cross-sectional study [49] was of moderate quality as study specific questions and not validate measures of willingness were used and there was a lack of clarity as to what were the eight questions used to assess willingness to use electronic devices (Appendix 3). The cohort study [50] was low to moderate in quality as it did not account for confounding in its study design, and it was unclear if appropriate follow-up of participants was conducted (Appendix 3).

### Barriers and facilitators

Barriers and facilitators to implementing digital psychosocial interventions designed for older adults in EDs were identified and mapped onto the TDF (Table 3). Three stakeholder groups were identified: older adults, family and caregivers, and healthcare professionals (HCPs). Across all three groups, barriers and facilitators were identified within the domains of ‘belief about consequences’ and ‘environmental context and resources’. Other elicited domains varied across stakeholder groups. Older adults identified the greatest number of barriers and facilitators, followed by HCPs and then family and caregivers.

**Table 3:** Barriers and facilitators to implementing digital tools for older adults in emergency departments

Stakeholder group	Older adults	Healthcare providers	Family and caregivers
**Knowledge (An awareness of the existence of something)**			
Barriers	• NR	• NR	• NR
Facilitators	• High level of understanding and comprehension of the psychosocial concerns [42]	• Certain staff (i.e. geriatricians) have the knowledge to assess psychosocial circumstances [51]	• NR
**Skills (An ability or proficiency acquired through practice)**			
Barriers	• Lack of technology skills [43, 48, 49]	• Difficulties identifying the need for psychosocial care [43, 51] • Training would be required to upskill most staff [51]	• NR
Facilitators	• Frequent/weekly use of technology outside of hospital [42, 48]	• Pre-existing skills using of technology in the ED [48]	• NR
**Social/professional role and identity (A coherent set of behaviours and displayed personal qualities of an individual in a social or work setting)**			
Barriers	• NR	• NR	• NR
Facilitators	• NR	• NR	• NR
**Beliefs about capabilities (Acceptance of the truth**,** reality**,** or validity about an ability**,** talent**,** or facility that a person can put to constructive use)**			
Barriers	• Lack of self-efficacy [42, 45, 48]	• Belief that older adults may not have the capability to perform screening [43, 51] • Belief that patients may lack knowledge to perform screening [43]	• Belief that older adults may not have the capability to perform screening [43, 45]
Facilitators	• Confidence in performing a self-assessment [42, 45, 49]	• NR	• NR
**Beliefs about consequences (Acceptance of the truth**,** reality**,** or validity about outcomes of a behaviour in a given situation)**			
Barriers	• Potential negative outcomes (i.e. mandatory reporting) of completing screening [43]	• Uncertainty about what happens following screening [43, 51]	• NR
Facilitators	• NR	• Help identify at risk patient [51]	• NR
**Reinforcement (Increasing the probability of a response by arranging a dependent relationship**,** or contingency**,** between the response and a given stimulus)**			
Barriers	• NR	• NR	• NR
Facilitators	• NR	• NR	• NR
**Intentions (A conscious decision to perform a behaviour or a resolve to act in a certain way)**			
Barriers	• NR	• NR	• NR
Facilitators	• NR	• NR	• NR
**Goals (Mental representations of outcomes or end states that an individual wants to achieve)**			
Barriers	• NR	• NR	• NR
Facilitators	• NR	• NR	• NR
**Memory**,** attention**,** and decision processes (The ability to retain information**,** focus selectively on aspects of the environment**,** and choose between two or more alternatives)**			
Barriers	• NR	• NR	• NR
Facilitators	• Ability to revisit the tool at multiple time points during an ED admission [42, 43]	• NR	• NR
**Environmental context and resources (Any circumstance of a person’s situation or environment that discourages or encourages the development of skills and abilities**,** independence**,** social competence**,** and adaptive behaviour)**			
Barriers	• Physical composition of digital device [48, 49] • The ED environment is uncomfortable [43]	• Time is required for appropriate screening [43, 51] • Standardised language can feel stilted [43] • Lack of information transfer [50, 51] • No specific process to identify older adults requiring psychosocial care [51]	• Accompanying screening of family and caregivers was too long [48] • Belief that ED is not the environment for screening [43]
Facilitators	• Appropriate older adult’s specific user experience design [42, 48] • Headphones helped Improved privacy [42, 43]	• Drop down menus and clear language [43, 48] • Tools already exist to screen for psychosocial problems [43, 51] • Quiet environment that aids in the delivery of screening [43]	• Possible preference for digital screening [45]
**Social influences (Those interpersonal processes that can cause individuals to change their thoughts**,** feelings**,** or behaviours)**			
Barriers	• Preference for interview style assessment over digital assessment [42, 45]	• NR	• NR
Facilitators	• Preference for self-assessment [42, 45]	• Positive attitude of senior management [51]	• NR
**Emotion (A complex reaction pattern**,** involving experiential**,** behavioural**,** and physiological elements**,** by which the individual attempts to deal with a personally significant matter or event)**			
Barriers	• Fear or dislike towards technology [42, 43, 45, 49] • Upset by psychosocial questioning [42, 43]	• Patients’ emotional attachment to their caregiver if that patient faced mistreatment from them [43]	• NR
Facilitators	• Positivity or support towards technology [42, 45, 48]	• Enjoyment of technology [48] • Empathy when discussing sensitive topics [43]	• NR
**Behavioural regulation (Anything aimed at managing or changing objectively observed or measured actions)**			
Barriers	• NR	• NR	• NR
Facilitators	• NR	• NR	• NR

NR = not reported

#### Older adults

##### Skills

The presence or absence of technology skills was identified as a significant barrier to use of a digital tool. A lack of skills was found to explain refusal by many prospective participants [49]. Participants who lacked technology skills felt less confident and comfortable using digital devices to enter health information [48, 49]: “If I’m not … savvy with computers and stuff, it’s harder on me” (Choo et al. (2021) [43]). However, participants who used technology in their day-to-day activities were more likely to agree to use a digital health intervention [48, 49]. Therefore, frequent previous computer or digital device use was a facilitator to implementing a digital health intervention in the ED.

##### Environmental context and resources

Aspects of digital devices used in ED identified as barriers and facilitators to implementation. Older adults identified several characteristics of digital health interventions that facilitate their use in EDs. Interventions that used user experience design methods were generally positively received by older participants. Their characteristics included large, easy-to-understand text, large buttons or stylus capabilities, and high-contrast colour schemes that accommodated reading ability, loss of dexterity and loss of visual acuity [42, 48]. The physical composition and functionality of the devices used to deliver the intervention were reported as both barriers and facilitators. In one study [48], the dimensions and weight of the tablet were barriers to implementation. When asked about their experience using the tablet, one participant in Tong and colleagues’ (2016) [48] study stated: “It’s heavy; make it lighter or have a book stand”. In contrast, devices such as iPads were identified as portable and lightweight and facilitated psychosocial screening in EDs [43]. The use of headphones on devices with read-aloud functions to enhance privacy and accommodate older adults with visual impairments or reading difficulties was found to facilitate implementation [42, 43]. 

##### Emotions

Older adults’ emotional response to technology or psychosocial questions was found to constitute a barrier [42, 43, 45, 48, 49]. Fear or dislike of technology was one of the major barriers to enrolment and to participants wanting to perform psychosocial screening using a digital device [45, 49]. In contrast, participants who had positive attitudes towards technology liked using digital psychosocial interventions in EDs and supported their implementation [42, 45, 49]. 

#### Healthcare providers

##### Belief about capabilities

The barriers identified by HCPs predominately aligned with the pejorative stereotype that older adults cannot use digital screening tools [43, 51]. These beliefs were influenced by factors such as the notion that the primary complaint prompting the older adult’s visit to the ED could hinder their ability to perform self-assessments, as well as concerns that older adults lack the knowledge or skills to complete screening via a tablet [43, 51]. One HCP in Choo and colleagues’ (2021) [43] qualitative study described their beliefs about older adults’ capabilities: “I think you would still have to have someone on the ground though. So, if I think of my mom, she would be like, ‘How do I do this? I don’t know. What’s this thing say? What is this pop-up?’”.

##### Environmental context and resources

HCPs identified various barriers related to the ED environment and resources. Barriers included workload, time, and the nature of tasks in ED. Although staff identified digital health interventions for older adults as potentially beneficial, the time required to perform the screening appropriately can be limited in EDs due to clinicians’ workloads and competing tasks [43, 50]. The throughput and workflows of the ED was identified as another barrier to the implementation of digital psychosocial interventions [43]. Patients in EDs require quick clinical assessments, with priority placed on patients’ primary complaints. This can prevent additional screening, such as of psychosocial needs, from occurring in EDs [43]. HCPs also said that a lack of information transfer among staff, departments and other hospitals could impede the implementation of digital health tools because implementation creates additional work for staff [50, 51]. 

#### Family and caregivers

##### Beliefs about capability

The views and perspectives of family members and caregivers mirrored the negative stereotype that older adults cannot perform digital screening. They identified various factors that potentially contribute to older adults’ difficulties using a digital psychosocial tool. These were the beliefs that older adults may not understand psychosocial needs and that this population lacks technology skills or may want nothing to do with technology [43, 45]. Families and caregivers stated that when asking an older adult to use the tool, if their primary complaint related to their physical health, this should be considered to ensure the older adult was physically able to use the tool [43, 45]. 

##### Environmental context and resources

On the whole, family and caregivers agreed with older adults that the ED was not the appropriate environment for psychosocial screening [43]. They suggested that locations such as a General Practitioner’s office would be more appropriate [43]. Additionally, when family and caregivers were asked to complete screening to assess the older adults they had accompanied to the ED, they felt it was too long [48]. In contrast, it was found in one study that family and caregivers rated digital assessments to be as acceptable as interview assessments [45]. 

##### Non-TDF themes

Age was the only barrier and facilitator that was inductively coded. Age younger than 85 years as a facilitator, whilst age older than 85 years was identified as a barrier [45, 49]. Older adults younger than 85 years, reported better performance on the digital tool [49, 50]; whilst adults older than 85 years were less likely to agree to use tablets or find the use of digital tools in the ED acceptable [45, 49]. Boucher et al. (2019) [45] also reported that adults older than 85 years had a significant preference for assessment performed by a research assistant rather than digital self-assessment. The only other study to report age as a possible barrier or facilitator, Abujarad et al. (2021) [42] found no difference by participant age in rating the usability of the digital tool.

## Discussion

### Principal findings

The findings of this review indicate that the implementation of digital psychosocial intervention in EDs is impacted by the behaviours and experiences of older adults, as well as their families, caregivers and HCPs.

The barriers and facilitators identified across the three stakeholder groups were mapped onto the following domains: ‘environmental context and resources’, ‘emotions’, ‘skills’, and ‘beliefs about capability’. The largest number of concerns were regarding the ability of older adults to use technology or digital devices. Some barriers were closely tied to elder abuse; it was thus difficult to assess whether these could be applied to digital psychosocial interventions more generally or if they relate only to interventions addressing sensitive topics.

Digital psychosocial tools for older adults are being implemented in emergency settings globally. The review findings suggest that although many barriers to implementation exist, these tools may aid in care provision [43, 51], improving older adults’ experiences in ED [42, 48], and overcoming barriers associated with the usual methods of care (i.e., paper- or interview-based screening) [43]. Recent decades have seen rapid developments in digital health interventions and increased implementation across health systems and populations [21]. This necessitates the need to ensure EDs and older adults are not forgotten, despite the implementation challenges highlighted in this review. Based the findings of this review and the continued developed of the digital health field, the evidence tends to support the development and implementation of digital psychosocial tools for older adults during an ED admission.

Many older adults reported positive attitudes towards using technology [42, 43, 45, 49]. Similar attitudes have been reported by older adults in other healthcare settings [52, 53]. These positive attitudes were more frequent in those younger than 85 years [45, 49]. Within the broader literature around older adults’ skills and abilities to use digital health technologies there are mixed results [25, 54–57]. Older studies report limited technological skills among this population [54]. However, as a result of the COVID-19 pandemic older adults were reported to rapidly adopt and adapted to the use of digital health technologies [57]. Research suggests that the digital divide within the population may continue to narrow, resulting in future generations of older adults having improved digital skills, facilitating the implementation of digital psychosocial tools in EDs [58]. 

However, the potential for improved skills cannot be the only mechanism that supports older adults to use technology in EDs. Digital psychosocial interventions can also be designed to account for the preferences and needs of older adults, such as declining dexterity, eyesight and cognitive function [42, 43, 45, 49]. Co-design can facilitate these changes during intervention creation. Digital psychosocial interventions should be designed to improve user experience and include the capacity for asynchronous use (the ability to revisit the technology several times to complete tasks) to continue promoting positive interactions between older adults and digital technology in EDs.

Crucially, there is a disconnect between the perspectives of older adults and other stakeholders [43, 45, 50]. Older adults reported feeling confident and willing to use technology, whereas HCPs, families, and caregivers assumed that older adults could not use digital tools [43, 45, 50]. Similar negative and ageist attitudes have been identified in a study examining HCPs’ attitudes in other healthcare settings [26]. These attitudes perpetuate pejorative stereotypes and potentially create barriers to implementation that would not otherwise exist. Research suggests that this belief may be linked to HCPs’ concerns that introducing technology into the ED may increase workloads and the time spent assisting patients in using digital psychosocial interventions [43, 50, 51]. However, incorporating digital health technologies into routine clinical care may improve resource allocation and the ability to follow-up patient care as well as patient experiences and outcomes [59]. Therefore, introducing digital health technologies into EDs may aid in creating supportive environment which could contribute to HCPs willingness to use them in the care of older adults. However, additional efforts must be made to change the attitudes of HCPs working in the ED to facilitate the implementation of digital psychosocial interventions.

### Implications for research

We have identified gaps in the current knowledge surrounding the implementation of digital psychosocial interventions for older adults in EDs. Various stakeholders’ perspectives, especially the perspectives of administrators (e.g., triage ward clerks) and non-clinical leaders (e.g., operations managers), are missing from the current research. Administrators and non-clinical leaders frequently have comprehensive oversight of ED and organisational systems, and how these would impact digital intervention implementation [30, 60]. 

The emergency department is a unique and complex environment for which customised and adaptable strategies are required to optimise uptake of new technologies [61]. Our research has highlighted the paucity of evidence surrounding sustained implementation of digital psychosocial intervention for older people in EDs. Future research should ensure inclusion of all stakeholder groups and use of sensitive, population appropriate methods to promote successful implementation. Additionally, this review has highlighted the importance of designing digital tools that cater to the needs of older adults, co-design provides an opportunity to facilitate the development of future tools.

### Strengths and limitations

To our knowledge, this is the first scoping review to identify and synthesise the barriers and facilitators to implementing digital psychosocial interventions for older adults in ED. The novel nature of this research area lends itself to a scoping review rather than a systematic review as the purpose of the review was to identify all the available evidence, key influences of implementation and knowledge gaps in this field [62]. The review was strengthened by the use of rigorous scoping review methods, including working with a specialist information analyst to develop our search strategy, following the JBI guidelines [33] and reporting the review using the PRISMA-ScR checklist [34]. Additionally, our use of the TDF to synthesise our findings allowed for investigation of barriers and facilitators from multiple perspectives. The TDF also allows for our findings to be used to develop behavioural interventions that target the older adults, family and caregivers, and HCPs working in ED.

There are some limitations to this review. Due to limited resources, only English-language publications were included. In addition, we did not search specific Information technology and computer science databases nor the reference lists of related systematic or scoping reviews, which may have resulted in missing potentially eligible papers. Furthermore, the complex and varied definitions of psychosocial well-being (definitions including a mixture of physical, economic, social, mental, emotional, cultural, and spiritual determinants of health [63]) may have resulted in overlooking papers reporting more specific components of psychosocial care or defining psychosocial care differently. However, working with a specialist information analyst and having a broad definition of psychosocial well-being was done to reduce the number of studies missed.

## Conclusions

The consideration of barriers and facilitators when designing digital health technologies for older adults in emergency settings is essential to ensuring their adoption and implementation. Findings from this review suggest successful implementation of digital health technologies in the ED can be optimised through ensuring older adults are given opportunity to engage with new technology without bias, provision of person-centred support as needed and sensitive design of digital tools adapted to specific needs of older people. Further research is required to better understand the perspectives of all stakeholders, including administrators, non-clinical leaders, ED clinicians, and families and caregivers of older adults in EDs.

## Supplementary Information

Below is the link to the electronic supplementary material.

Supplementary Material 1

Supplementary Material 2

Supplementary Material 3

## Data Availability

Not applicable. All data included in this review is publicly available.

## Abbreviations

CASP: Critical Appraisal Skills Programme
ED: Emergency department
HCP: Healthcare provider
JBI: Joanna Briggs Institute
PRISMA-ScR: Preferred Reporting Items for Systematic reviews and Meta-Analyses extension for scoping reviews
SPIDER: Sample, phenomenon of interest, design, evaluation, research type
TDF: Theoretical domains framework
RCT: Randomised controlled trial
