# Supplementary material for: Barriers and facilitators to implementing digital psychosocial interventions for older adults presenting to emergency departments: a scoping review
Source: BMC Health Serv Res. 2026 Feb 19;26:402. doi: 10.1186/s12913-026-14129-6 (PMC13020092; doi:10.1186/s12913-026-14129-6)
Supplement: Supplementary file 1 — Supplementary Material 1 [file 12913_2026_14129_MOESM1_ESM.docx]

Appendix 1

Medline (OVID) Search strategy

1. emergency service, hospital/ or trauma centers/
2. (emergency service or trauma cent* or emergency department* or emergency setting* or emergency room* or Emergency unit* or Emergency ward* or "accident and emergency department*" or "A and E department*" or emergency care or "A and E unit" or "A and E ward" or triage).mp.
3. (casualty adj (department* or ward* or unit* or room*)).mp.
4. (ED adj (care or service or setting or visit? or presentation* or attendance* or admission*)).mp.
5. 1 or 2 or 3 or 4
6. mobile applications/ or user-computer interface/ or information technology/ or digital technology/ or Computers, Handheld/ or smartphone/ or Cell Phone/ or Web Browser/
7. ((user* adj1 computer interface*) or information technol* or (digital adj2 technol*) or (digital adj2 intervention*) or digital health*).mp.
8. ((ehealth or mhealth or "e health" or "m health") adj (intervention* or tool?)).mp.
9. web-based intervention.mp.
10. (online adj (intervention* or tool* or screening)).mp.
11. ((mobile or smartphone or smart phone or cell phone or tablet or mhealth or ehealth or mobile phone or iphone or ipad or health) adj3 (app or apps or application*)).mp.
12. (digital tool? or digital screening or electronic screening or interactive tool? or interactive screen? or electronic tool? or audiovisual medium or audiovisual tool? or audio-visual medium or audio-visual tool? or smart device* or tablet computer* or handheld computer* or hand-held computer* or handheld digital or hand-held digital).mp.
13. (patient facing or patient-facing or patient portal*).mp.
14. (palm pilot? or palm-top computer* or palm-held computer* or palm-held digital device* or personal digital assistant or portable electronic app? or portable electronic application* or portable software app? or portable software application*).mp.
15. 6 or 7 or 8 or 9 or 10 or 11 or 12 or 13 or 14
16. aged/ or "aged, 80 and over"/ or centenarians/ or nonagenarians/ or octogenarians/
17. ("aged 80 and over" or centenarian* or nonagenarian* or octogenarian* or septuagenarian* or senior citizen* or old* adult* or old* people or old* person* or old* patient* or old* citizen* or old* population* or old* individual*).mp.
18. (elderly or geriatric* or gerontol* or old age).mp.
19. (70+ years or 75+ years or 80+ years or 85+ years or 90+ years or 95+ years or 100+ years or 70 plus or 75 plus or 80 plus or 85 plus or 90 plus or 95 plus or 100 plus).mp.
20. (adult? aged over adj ("70" or "75" or "80" or "85" or "90" or "95" or "100")).mp.
21. (adult? over adj (seventy or seventy five or eighty or eighty five or ninety or ninety five or one hundred)).mp.
22. (adult? aged adj (seventy or seventy five or eighty or eighty five or ninety or ninety five or one hundred)).mp.
23. (aged 70 or aged 75 or aged 80 or aged 85 or aged 90 or aged 95 or aged 100).mp.
24. (("70" or "75" or "80" or "85" or "90" or "95" or "100") adj ("years of age" or "years old")).mp.
25. 16 or 17 or 18 or 19 or 20 or 21 or 22 or 23 or 24
26. 5 and 15 and 25
