# Supplementary material for: Barriers and facilitators to implementing digital psychosocial interventions for older adults presenting to emergency departments: a scoping review
Source: BMC Health Serv Res. 2026 Feb 19;26:402. doi: 10.1186/s12913-026-14129-6 (PMC13020092; doi:10.1186/s12913-026-14129-6)
Supplement: Supplementary file 2 — Supplementary Material 2 [file 12913_2026_14129_MOESM2_ESM.docx]

Appendix 2

Excluded Studies

| **Study** | **Reason for exclusion** |
| --- | --- |
| Abou-Abdallah M, Lamyman A. Exploring communication difficulties with deaf patients. Clinical Medicine, Journal of the Royal College of Physicians of London. 2021;21(4). PMID: 2013925325. | Wrong intervention |
| Abujarad F, Choo E, Pantalon MV, Jubanyik K, Dziura J, Edwards C, et al. Integrating a Digital Elder Mistreatment Intervention in the Emergency Department Setting. Acad Emerg Med. 2023;30(Supplement 1):211. PMID: 641605750 | Conference abstract |
| Abujarad F, Edwards C, Choo E, Gill T. Screening for elder abuse with digital health technology. J Am Geriatr Soc. 2021;69(SUPPL 1):S87. PMID: 634827295. | Conference abstract |
| Abujarad F, Edwards C, Choo E, Pantalon M, Jubanyik K, Dziura J, et al. Digital health screening tool for identification of elder mistreatment. Gerontechnology : international journal on the fundamental aspects of technology to serve the ageing society. 2020;19(Suppl 1). | Conference abstract |
| Adam HD, Robert B, Shamis N, Desmond ON, Desmond ON, Sean K. The Abbreviated Mental Test 4 for cognitive screening of older adults presenting to the Emergency Department. 2017. | Wrong intervention |
| Albrecht R, Espejo T, Riedel HB, Nissen SK, Banerjee J, Conroy SP, et al. Clinical Frailty Scale at presentation to the emergency department: interrater reliability and use of algorithm-assisted assessment. Eur Geriatr Med. 2024;15(1):105-13. | Wrong outcomes |
| Amy CC, Amy C, Karthik A, Amro K, Daniel RR, Lukasz M, et al. Codesign approaches involving older adults in the development of electronic healthcare tools: a systematic review. 2022. | Wrong setting |
| Appel L, Kisonas E, Appel E, Klein J, Bartlett D, Rosenberg J, et al. Introducing virtual reality therapy for inpatients with dementia admitted to an acute care hospital: learnings from a pilot to pave the way to a randomized controlled trial. Pilot and Feasibility Studies. 2020;6(1):166. PMID: 2007131340. | Wrong setting |
| Aranha M, Shemie J, James K, Deasy C, Heavin C. Behavioural intention of mobile health adoption: A study of older adults presenting to the emergency department. Smart Health. 2024;31((Aranha, Shemie, Deasy) School of Medicine, University College Cork, Cork, Ireland(James) Consultant in Geriatric Medicine, Cork University Hospital, Cork, Ireland(Deasy) Emergency Medicine, Cork University Hospital, Cork, Ireland(Heavin) Business Informa):100435. PMID: 2028772736. | Wrong interventions |
| Armin N, Jeff D, Jeffrey D, William P, Mary S, Amy A, et al. Detecting Cognitive Impairment and Dementia in the Emergency Department: A Scoping Review. 2022. | Wrong patient population |
| Barton HJ, Salwei ME, Rutkowski RA, Wust K, Krause S, Hoonakker PL, et al. Evaluating the Usability of an Emergency Department After Visit Summary: Staged Heuristic Evaluation. JMIR human factors. 2023;10(101666561):e43729. | Wrong study design |
| Bhattacharyya O, Mossman K, Gustafsson L, Schneider EC. Using human-centered design to build a digital health advisor for patients with complex needs: persona and prototype development. J Med Internet Res. 2019;21(5):e10318. | Wrong setting |
| Botella C, Etchemendy E, Castilla D, Baños RM, García-Palacios A, Quero S, et al. An e-health system for the elderly (Butler Project): A pilot study on acceptance and satisfaction. CyberPsychology & Behavior. 2009;12(3):255-62. | Wrong setting |
| Boucher V, Lamontagne M, Lee J, Emond M. Feasibility of self-assessing functional status in older emergency department patients. Canadian Journal of Emergency Medicine. 2020;22(Supplement 1):S111. PMID: 633284431. | Conference abstract |
| Brahmandam S, Holland WC, Mangipudi S, Braz VA, Medlin R, Jones CW, et al. Willingness and ability of older emergency department patients to provide clinical information using a tablet computer. Acad Emerg Med. 2015;22(5 SUPPL. 1):S193. PMID: 71879091. | Conference abstract |
| Brahmandam S, Holland WC, Mangipudi S, Braz VA, Medlin R, Jones CW, et al. Willingness and ability of older emergency department patients to provide clinical information using a tablet computer. J Am Geriatr Soc. 2015;63(SUPPL. 1):S101-S2. PMID: 71856031. | Conference abstract |
| Bunney G, Tran S, Han S, Gu C, Wang H, Luo Y, et al. Using Machine Learning to Predict Hospital Disposition With Geriatric Emergency Department Innovation Intervention. Ann Emerg Med. 2023;81(3):353-63. | Wrong intervention |
| Carpenter CR, Leggett J, Bellolio F, Betz M, Carnahan RM, Carr D, et al. Emergency Department Communication in Persons Living With Dementia and Care Partners: A Scoping Review. J Am Med Dir Assoc. 2022;23(8):1313.e15-.e46. PMID: 2019599886. | Wrong intervention |
| Choo EK, Edwards C, Abuwandi M, Carlson K, Bonito J, Jubanyik K, et al. Perceptions of older adults and health professionals about digital health tools for elder mistreatment screening. Acad Emerg Med. 2021;28(SUPPL 1):S234. PMID: 635077100. | Conference abstract |
| Conroy SP, Ansari K, Williams M, Laithwaite E, Teasdale B, Dawson J, et al. A controlled evaluation of comprehensive geriatric assessment in the emergency department: the ‘Emergency Frailty Unit’. Age Ageing. 2014;43(1):109-14. | Wrong intervention |
| Genes N. mHealth in emergency medicine. Emerg Med Pract. 2017 (Suppl 2017A):1-11. | Wrong patient population |
| Graham E, Marshall T, Claire R, Trudi M. Comprehensive geriatric assessment in the emergency department. 2014. | Wrong intervention |
| Jesus M, Cagigal C, Silva T, Martins V. Fronto-temporal dementia and mania: The problem of the differential diagnosis. Eur Neuropsychopharmacol. 2019;29(Supplement 1):S158-S9. PMID: 2001445402. | Conference abstract |
| John M, Sean K, John JM. Acute care of older patients in the emergency department: strategies to improve patient outcomes. 2015. | Wrong outcomes |
| Jones BA, Chu DI. Impact of an automated peri-procedural digital health intervention on rates of emergency department visits and readmissions. The American Journal of Surgery. 2023;226(5):596-7. | Wrong study design |
| Jubanyik KJ, Choo E, Pantalon MV, Dziura J, Edwards C, Gill T, et al. 404 Emergency Department Tablet-Based Screening Tool for Elder Abuse. Ann Emerg Med. 2020;76(4 Supplement):S154. PMID: 2008409806. | Conference abstract |
| Lee JS, Tong T, Tierney MC, Kiss A, Chignell M. Predictive Ability of a Serious Game to Identify Emergency Patients With Unrecognized Delirium. J Am Geriatr Soc. 2019;67(11):2370-5. | Wrong outcomes |
| Liu Z, Ng M, Gunasekeran DV, Li H, Ponampalam K, Ponampalam R. Mobile technology: Usage and perspective of patients and caregivers presenting to a tertiary care emergency department. World J Emerg Med. 2020;11(1):5. | Wrong patient population |
| MacDonald L-Q, Berman L. Accommodations and Interventions to Decrease Unnecessary ED Utilization in Patients with Limited English Proficiency. Journal of Technology in Behavioral Science. 2021;6(1):74-80. | Wrong patient population |
| McFeely A, Corcoran R, Jusmanova K, Kearns R, Marks E, Ward L, et al. Geriatric emergency services (GEMS): An acute floor frailty service model. Age Ageing. 2018;47(Supplement 5). PMID: 627247578. | Conference abstract |
| Nana A, Carla L. Identification of seniors at risk (ISAR) screening tool in the emergency department: implementation using the plan-do-study-act model and validation results. 2014. | Wrong study design |
| Østervang C, Jensen CM, Coyne E, Dieperink KB, Lassen A. Usability and Evaluation of a Health Information System in the Emergency Department: Mixed Methods Study. JMIR Hum Factors. 2024;11(1). | Wrong patient population |
| Ouchi K, Knabben V, Rivera-Reyes L, Gan ta N, Gelfman LP, Sudore R, et al. Preparing Older Adults with Serious Illness to Formulate Their Goals for Medical Care in the Emergency Department. J Palliat Med. 2017;20(4):404-8. PMID: 615273836. | Wrong study design |
| Post L, Conner T, Oehmke J, Abujarad F, Leo C, Brandt C, et al. Development and Validation of the Emergency Department Geriatric Readmission Assessment at Yale (ED GRAY): Part 2, Prognostic Accuracy. Br J Med Med Res. 2016;14:1-8. | Wrong outcomes |
| Post L, Conner T, Oehmke J, Abujarad F, Leo C, Brandt C, et al. Development and Validation of the Emergency Department Geriatric Readmission Assessment at Yale (ED GRAY): Part 1, Fundamental Measurement. Br J Med Med Res. 2016;14:1-14. | Wrong study design |
| Post LA, Dziura J, Brandt C, D'Onofrio G, Ulrich A, Cooney L, et al. A brief assessment and prognosis of cognitive and physical disabilities for elderly ed patients. Acad Emerg Med. 2016;23(SUPPL. 1):S101. PMID: 72280913. | Conference abstract |
| Preschl B, Wagner B, Forstmeier S, Maercker A. E-health interventions for depression, anxiety disorders, dementia, and other disorders in old age: A review. Journal of CyberTherapy and Rehabilitation. 2011;4:371-85. | Wrong setting |
| Rosen CB, Roberts SE, Syvyk S, Finn C, Tong J, Wirtalla C, et al. A Novel Mobile App to Identify Patients With Multimorbidity in the Emergency Setting: Development of an App and Feasibility Trial. JMIR formative research. 2023;7(101726394):e42970. | Wrong study design |
| Shagerdi G, Ayatollahi H, Hemmat M. Emergency care for the elderly: A review of the application of health information technology. Health Policy and Technology. 2022;11(1):100592. PMID: 2015912873. | Wrong study design |
| Silva S, Felgueiras R, Oliveira IC. Geriatric helper: an mHealth application to support comprehensive geriatric assessment. Sensors. 2018;18(4):1285. | Wrong setting |
| Sukkird V, Shirahada K. Technology challenges to healthcare service innovation in aging Asia: Case of value co-creation in emergency medical support system. Technology in Society. 2015;43:122-8. | Wrong study design |
| Sukkird V, Shirahada K. E-health service model for Asian developing countries: A case of emergency medical service for elderly people in Thailand. IGI Global; 2018. p. 214-32. | Wrong study design |
| Tandon U, Ertz M, Shashi. Continued Intention of mHealth Care Applications among the Elderly: An Enabler and Inhibitor Perspective. International Journal of Human–Computer Interaction. 2023:1-16. | Wrong setting |
| Theodore DC, Joseph F, Ipsit VV, Andrew S, John T. Mobilizing mHealth Data Collection in Older Adults: Challenges and Opportunities. 2019. | Wrong intervention |
| Tong T, Chignell M, Tierney MC, Lee J. A Serious Game for Clinical Assessment of Cognitive Status: Validation Study. JMIR serious games. 2016;4(1):e7. | Wrong study design |
| Tony R, Michael ES, Alyssa E, Mary RM. Identifying and initiating intervention for elder abuse and neglect in the emergency department. 2018. | Wrong intervention |
| Wilkinson A, Tong T, Zare A, Kanik M, Chignell M. Monitoring Health Status in Long Term Care Through the Use of Ambient Technologies and Serious Games. IEEE journal of biomedical and health informatics. 2018;22(6):1807-13. | Wrong outcomes |
