# Supplementary material for: Barriers and facilitators to implementing digital psychosocial interventions for older adults presenting to emergency departments: a scoping review
Source: BMC Health Serv Res. 2026 Feb 19;26:402. doi: 10.1186/s12913-026-14129-6 (PMC13020092; doi:10.1186/s12913-026-14129-6)
Supplement: Supplementary file 3 — Supplementary Material 3 [file 12913_2026_14129_MOESM3_ESM.docx]

Appendix 3

Quality assessment for each article

Appendix 3 Table 1. Critical appraisal for qualitative and mixed-methods studies

| Article | Abujarad et al. (2021) | Choo et al. (2021) | Shagerdi et al. (2022) | Tong et al. (2016) |
| --- | --- | --- | --- | --- |
| Clear aims | Yes | Yes | Yes | Yes |
| Appropriate methodology | Yes | Yes | Yes | Can't tell |
| Appropriate design | Yes | Yes | Yes | No |
| Appropriate recruitment strategy | Yes | Yes | No | Can't tell |
| data collection | Yes | Yes | No | Yes |
| relationship between researcher and participant | Yes | Yes | No | Yes |
| No Ethical issues | Yes | Yes | Yes | Yes |
| rigorous data analysis | No | Yes | Yes | Yes |
| clear findings statement | Yes | Yes | Yes | Yes |

Appendix 3 Table 2. Critical appraisal for randomised control trials

| Article | Boucher et al. (2019) |
| --- | --- |
| Clear research question | Yes |
| Randomisation | Yes |
| Participants accounted for | Yes |
| Participant blinding | No |
| Assessor blinding | No |
| Similar participant characteristics | Yes |
| same care between groups | Can't tell |
| Effects reported | Yes |
| precision in reporting results | Yes |
| Benefits outway harms | Yes |
| local context application | No |
| Value of intervention | Yes |

Appendix 3 Table 3. Critical appraisal for cross-sectional studies

| Article | Brahmandam et al. (2016) |
| --- | --- |
| Did the study address a clearly focused issue? | Yes |
| Did the authors use an appropriate method  to answer their question? | Yes |
| Were the subjects recruited in an acceptable way? | Yes |
| Were the measures accurately measured to reduce bias? | No |
| Were the data collected in a way that addressed the research issue? | Yes |
| Did the study have enough participants to minimise the play of chance? | Yes |
| How are the results presented and what is the main result? | Yes |
| Was the data analysis sufficiently rigorous? | Yes |
| Is there a clear statement of findings? | Can’t Tell |
| Can the results be applied to the local population? | No |
| How valuable is the research? | Can’t Tell |

Appendix 3 Table 4. Critical appraisal for cohort studies

| Article | Saario et al. (2021) |
| --- | --- |
| Clear issue | Yes |
| Acceptable recruitment | Yes |
| accurately measure exposure | Yes |
| accurately measured outcome | Yes |
| identified confounding | Yes |
| confounding in design, analysis | No |
| follow up | Can't tell |
| follow up-long enough? | Can't tell |
